# Editorial: Using Artificial Intelligence to Enhance Sport Performance

**DOI:** 10.3389/fspor.2022.886730

**Published:** 2022-04-25

**Authors:** Dan B. Dwyer, Matthias Kempe, Arno Knobbe

**Affiliations:** ^1^School of Exercise and Nutrition Sciences, Deakin University, Geelong, VIC, Australia; ^2^Department for Human Movement Sciences, University of Groningen, Groningen, Netherlands; ^3^Leiden Institute of Advanced Computer Science, Universiteit Leiden, Leiden, Netherlands

**Keywords:** sport, artificial intelligence, performance, machine learning, decision making

The purpose of this special topic is to provide an opportunity for researchers and practitioners to showcase the most recent advances in machine learning and artificial intelligence in sport. The relatively recent expansion of technology in sport has enabled an explosion in the amount of data collected and also the range and variety of attributes that are measured and recorded. This tsunami of data has created both a burden and an opportunity to answer some critically important questions that are faced by coaches and athletes. How can I train more effectively, how can I be more competitive and how can I avoid injury? In many cases the applicability of traditional statistical techniques has been exhausted and machine learning methods have been applied, adapted and developed, to analyse sport data.

In 1995, Lapham and Bartlett described artificial intelligence (AI) as holding the potential to support and improve decision-making in sports and speed up the analytic process to free up time and resources for experts. The use of Machine Learning (ML) has been advocated since then to build such decision support systems (Robertson, 2020). ML can be applied in sport to create many benefits including; to automate or semi-automate the collection of data, to (pre-)process data into meaningful information, to understand what information is important with respect to health and performance, and finally, to help coaches and athletes make complex decisions. Elite coaches and athletes frequently use their experience, knowledge and intuition to make successful decisions. However, some critical decisions are very difficult because the number of factors to consider and their interactions are too complex. Where the right kind of data are available, Machine Learning methods can be used to create models that can support complex decision-making.

Many of the articles in this Research Topic represent the adaptation of existing, and the development of novel ML methods, that solve real and significant problems in sport and confer many of the benefits mentioned above. In terms of improving the gathering and processing of data, Hosp et al. created a deep learning tool to automatically classify the level perceptual performance expertise of football (soccer) goalkeepers, while van Dijk et al. described how their advancements on the processing of data from inertial measurement units, produces more accurate information about body position in wheelchair sports. Furthermore, Schmid et al. created a tool to automatically identify tactical pattern-based player trajectories in American Football and used these automated annotations to create a simulation for defensive schemes and performance.

The development of new measures to evaluate individual and team performance was the main point of the majority of the studies in this Research Topic. Anzer and Bauer presented an improved method for calculating expected goals (i.e., scoring probability) in football (soccer) which is a well established measure of individual and team performance. The concept of “zones of control” is an important concept in the analysis and understanding of tactical performance in invasion sports such as football. Martens et al. described a more advanced method to calculate the control of space which has the potential to improve the way offensive performance is analyzed. This idea was closely linked to Dick et al. who explored ball possession phases in football in order to better understand how player behavior determines the outcome of the phase. They presented a new set of performance indicators that can be used to coach and assess player performance.

The last group of papers created models to predict athletes' performance, including Olthof et al. who explored the complex relationship between biomechanical load and performance in basketball players before and during games. They revealed that game load is a good predictor of technical performance and training load 2 days before gameday is a good predictor of game load. Finally, Kholkine et al. developed a method to help coaches understand who the main adversaries would be in 1-day road cycling races. Their prediction model can also help identify the important factors for winning a specific race and adapt the strategy of the team if certain riders display patterns of possibly winning a future race.

Every article in this Research Topic represents a valuable advancement in ML in sport. Collectively, the findings of the articles improve how we process raw data, clarify which variables represent the most important indicators of performance and provide prediction models that can be used to support complex decision making. The application of ML in sport has been justified based on what it can offer sport. However, we may soon reach the conclusion that sport offers something uniquely valuable to Machine Learning. Sport can provide high quality, rich data sets about many different human behaviors (e.g., movement, actions, performance, interactions, health, etc.) from a variety of different contexts. The behavior and performance of athletes are constrained by clearly-defined rules and trained experts can establish ground truths to support supervised Machine Learning. Many of these characteristics do not apply in a business context where much of the development of Machine Learning occurs.

Our hope is that the development of Machine Learning in sport continues, by educating students and practitioners about its benefits and applicability to sport. Indeed, each field (sport and data science) is sufficiently complex, and it may be wise to move ahead by creating partnerships between Sport Scientists and Data Scientists. We need the leaders in this field to continue to drive innovation but also to provide guidance toward the most beneficial solutions and away from ethically risky applications. Finally, we look forward to seeing the machine learning solutions that have been developed, deployed in the field to work for us by collecting data, conducting preliminary analyses and making recommendations.

## Author Contributions

DD wrote the first draft, then MK and AK edited this draft to create the final version. All authors designed the structure and content of the editorial.

## Conflict of Interest

The authors declare that the research was conducted in the absence of any commercial or financial relationships that could be construed as a potential conflict of interest.

## Publisher's Note

All claims expressed in this article are solely those of the authors and do not necessarily represent those of their affiliated organizations, or those of the publisher, the editors and the reviewers. Any product that may be evaluated in this article, or claim that may be made by its manufacturer, is not guaranteed or endorsed by the publisher.
